# Assessing the Clinical Utility of Multimodal Large Language Models in the Diagnosis and Management of Pigmented Choroidal Lesions

**DOI:** 10.1167/tvst.14.10.13

**Published:** 2025-10-14

**Authors:** Nehal Nailesh Mehta, Evan Walker, Elena Flester, Gillian Folk, Akshay Agnihotri, Ines D. Nagel, Melanie Tran, Michael H. Goldbaum, Shyamanga Borooah, Nathan L. Scott

**Affiliations:** 1 Jacobs Retina Center, Shiley Eye Institute, University of California, San Diego, La Jolla, CA, USA; 2 Viterbi Family Department of Ophthalmology and Shiley Eye Institute, University of California, San Diego, La Jolla, CA, USA; 3 School of Medicine, University of California, San Diego, La Jolla, CA, USA

**Keywords:** nevus, melanoma, MLLM

## Abstract

**Purpose:**

To evaluate the diagnostic and treatment recommendation performance of multimodal large language models (MLLMs) in identifying and classifying retinal lesions as choroidal nevus or melanoma, as well as compare their performance with expert human graders.

**Methods:**

This retrospective cross-sectional study included 48 eyes from 47 patients diagnosed with either choroidal nevus or melanoma. Patient demographics, including age, sex, ethnicity, best-corrected visual acuity (BCVA), and symptoms, were documented. Color fundus, autofluorescence, optical coherence tomography, and B-scan images were collected. The ocular images and patient characteristics were presented to ChatGPT 4.0, Gemini Advanced 1.5 Pro, and Perplexity Pro. Responses were recorded and compared with the clinical diagnoses and treatment recommendations made by two expert human graders. Diagnostic and treatment agreement, accuracy, sensitivity, and specificity were analyzed.

**Results:**

Gemini consistently outperformed ChatGPT and Perplexity across diagnostic and treatment prompts. The highest model performance was observed for prompts requesting treatment recommendations with clinical information, where Gemini achieved the highest accuracy (0.725), followed by Perplexity (0.647) and ChatGPT (0.314). Performance was lowest for prompts requiring strict clinical criteria, with all models showing poor sensitivity. Both human graders outperformed all MLLMs in accuracy and sensitivity on most prompts (*P* < 0.005). Accuracy did not improve when provided demographic or clinical data, except for Gemini.

**Conclusions:**

Human graders outperform current MLLMs, which show only moderate ability to diagnose choroidal nevi or melanoma from imaging.

**Translational Relevance:**

This study highlights limitations and potential of MLLMs in aiding diagnosis and treatment of choroidal lesions.

## Introduction

Recent advances in artificial intelligence have produced a range of language models (LLMs), including ChatGPT by OpenAI, Gemini by Google DeepMind, and Perplexity by Perplexity AI, that are designed to understand and generate human-like text from diverse inputs. Research in the medical literature has explored the potential of these artificial intelligence (AI) systems for various applications, including supporting clinical decision-making,^1^ delivering patient education with clear explanations,^2^ streamlining clinical documentation to reduce administrative burdens,^3^^,^^4^ synthesizing findings from vast bodies of literature,^5^ and enhancing medical training through interactive, simulation-based scenarios.^6^ In ophthalmology, AI and LLMs have been increasingly applied for tasks such as automated image interpretation, screening for retinal diseases, predicting disease progression, and assisting clinical decision-making, showing promise in enhancing patient care and workflow efficiency.^7^^–^^9^

In 2023, a major advancement came with ChatGPT-4, which introduced multimodal analysis capabilities, including medical imaging. This development has led to a new generation of models called multimodal large language models (MLLMs), such as Claude (Anthropic), Gemini (Google DeepMind), and Perplexity (Perplexity AI), that integrate natural language understanding with image processing. Given these recent capabilities, it is logical to consider the utility of AI in resolving complex diagnostic challenges. An example, in ophthalmologic imaging, is the differentiation between choroidal nevus and melanoma. Choroidal nevi are a common incidental finding observed in approximately 4.7% of the adult US population, and their prevalence increases with age.^10^ Although typically benign, a choroidal nevus is sometimes difficult to distinguish from choroidal melanoma. Choroidal melanomas are malignant lesions characterized by aggressive growth and a propensity for metastasis, meaning that it is critical for optometrists and ophthalmologists to correctly differentiate melanomas from nevi.^10^^,^^11^ To add a layer of complexity, studies suggest that around 1 in 8845 choroidal nevi transform into melanoma each year.^10^ There are now established criteria to guide ophthalmologists in the diagnosis of choroidal melanoma developed from large multicenter studies such as the Collaborative Ocular Melanoma Study (COMS) and modified using more recent studies.^12^^–^^14^ Criteria include lesion diameter greater than 5 mm, typically assessed using ultra-widefield pseudocolor fundus photography; the presence of orange pigment, detectable on fundus autofluorescence; the presence of subretinal fluid overlying the lesion, visualized by optical coherence tomography (OCT); lesion thickness greater than 2 mm, measured by B-scan ultrasound; and acoustic hollowness on B-scan ultrasound.^15^^,^^16^

Our study aims to compare the accuracy of these MLLMs when analyzing retinal imaging for eyes exhibiting either choroidal nevus or melanoma. To understand the clinical utility of the application of MLLMs in clinical settings, we compare the findings against those of ocular oncology specialists.

## Methods

This cross-sectional study was conducted at the Department of Ophthalmology, University of California, San Diego (UCSD) as a retrospective analysis of data from October 12, 2022, to September 11, 2024. The study was approved by the Institutional Review Board of UCSD, which deemed that individual patient consents were not required due to the retrospective nature of the study. However, patients had consented to imaging and the use of clinical data by institutional policy. The study met the ethical standards in the Declaration of Helsinki and the Health Insurance Portability and Accountability Act of 1996. All patient data were anonymized and had any metadata removed.

Patients were identified using the in-built search function on the electronic records (EPIC, Verona, WI, USA). A search was conducted for records between April 2019 and September 2024 by using the search terms “choroidal nevus” and “choroidal melanoma.” Candidate cases were reviewed manually, and corresponding ocular images were obtained. Inclusion criteria were the presence of a pigmented choroidal lesion, diagnosed as either a choroidal nevus or a melanoma, and availability of sufficient ocular imaging. Melanoma cases were included only if there was biopsy confirmation of the diagnosis. Nevus cases were included if the lesion remained stable on imaging over a minimum follow-up period of 6 months, with no signs of growth or malignant transformation. Only treatment-naive eyes were included. Eyes without biopsy-proven melanoma or without sufficient follow-up to confirm nevus stability were excluded.

For each patient, key demographic and clinical data, including age, sex, ethnicity, best-corrected visual acuity (BCVA), and presenting symptoms, were recorded. Color and autofluorescence retinal images were obtained using the OPTOS system (Optos PLC, Dunfermline, Scotland). Spectral-domain OCT (SD-OCT) images were captured with the Spectralis SD-OCT (Heidelberg Engineering, Heidelberg, Germany), and ultrasound B-scan images were acquired using the Quantel Medical Aviso B-Scan Ultrasound system (Cournon-d'Auvergne, Auvergne-Rhône-Alpes, France).

Ocular images and prompts were evaluated by three MLLMs, including ChatGPT-4 (OpenAI, San Francisco, CA, USA), Google Gemini 1.5 Pro (Google LLC, Mountain View, CA, USA), and Perplexity Pro (Perplexity AI, San Francisco, CA, USA), and two human graders (NLS and MHG). Perplexity is a platform that integrates multiple underlying LLMs, including models such as GPT-4.1, Gemini, and Grok. It has also developed its own “Sonar” models, which are based on architectures such as Llama 3.3 or DeepSeek. For this study, Perplexity Pro was used in its default configuration during the study period (November–December 2024), which employed the Llama 3.1 Sonar model as its underlying engine. For all the MLLMs, the ocular images and associated patient characteristics were presented via their respective temporary chat interfaces. To prevent ChatGPT-4o from learning from previous inputs, a new chat with the “Temporary Chat” function was used for each prompt, along with its corresponding set of images, ensuring that chat history was cleared between prompts. In Perplexity Llama 3.1 Sonar, this was achieved by disabling “AI Data Retention.” In Google Gemini 1.5 Pro, history was deleted manually before moving on to the next prompt. Ocular image prompting into MLLMs was done between November 1, 2024, and December 1, 2024.

Ground-truth diagnosis was decided on biopsy results for melanoma and stability on imaging interpretation for choroidal nevi. Lesions diagnosed by imaging interpretation were categorized as a choroidal nevus if the lesion showed no progression over at least 6 months of follow-up with ocular imaging (including color, autofluorescence, SD-OCT, and ultrasound B-scan).

A total of six diagnostic and treatment prompts were administered to assess diagnostic accuracy and decision-making in a stepwise manner to understand the effects of the different elements on MLLM decision-making. The prompts were as follows: prompt 1 (multiple-choice diagnosis), prompt 2 (melanoma vs. nevus), prompt 3 (melanoma vs. nevus with clinical information), prompt 4 (COMS criteria), prompt 5 (treatment), and prompt 6 (treatment with clinical information). The details of the prompts are presented in the supplementary data. All prompts were designed using recommendations for prompt engineering and prefaced with an opening statement (“Answer the following question as an ophthalmologist.”) to cue MLLMs to not assume any other background information unless given.^17^^,^^18^

Two expert human ocular oncology graders (NLS and MHG) independently reviewed the same ocular images and were provided the same diagnostic and treatment prompts as the MLLMs. All images were randomized and anonymized. Unlike the MLLMs, which were evaluated using temporary chat interfaces to prevent memory bias, the human graders had no such constraint. To mitigate the potential bias of knowing that all images represented either a choroidal nevus or melanoma, we introduced additional images of unrelated ocular pathology. These cases were not included in the statistical analysis and served only to discourage learned binary selections in prompt 1.

The human grading process was staged. Initially, graders were presented with the full image set and completed prompt 1. Following this, they received the same set of images and responded to prompts 2, 4, and 5. Clinical information, including age, sex, ethnicity, BCVA, and presenting symptoms, was then provided, after which the graders reevaluated the same images for prompts 3 and 6. Cases were randomized for each prompt, and graders were not allowed to return to previous prompts to alter responses. Intergrader agreement was assessed using Cohen's κ coefficient.

### Statistical Analysis

Subject-level demographic information is displayed as count (%) and mean (95% confidence interval [CI] for categorical and continuous parameters, respectively. Human grader and MLLM performance were assessed per prompt using accuracy, sensitivity, and specificity. A clustered bootstrap random sampling process, in which sampling was conducted at the subject level, was utilized to produce 95% CI estimates per performance metric. Performance metrics were also compared between MLLMs, per prompt, using the bootstrap random sampling process. A Bonferroni correction was applied to account for multiple comparisons across prompts and human grader and MLLM models, adjusting the significance threshold accordingly. All statistical analyses were conducted using the R programming language for statistical computation, version 4.4.0 (R Core Team, R Foundation for Statistical Computing, Vienna, Austria). An error analysis in the form of misclassification analysis was added in the end for MLLMs so we could assess what kind of diagnoses or decisions were made compared to the ground truth to understand if certain systematic decision errors were being made.

## Results

### Cohort Characteristics

A total of 48 eyes from 47 patients were included in the study. The mean age of patients was 65.5 years (95% CI, 62.3–68.8), with 54% of participants being female. The ethnic distribution was predominantly white (74.0%), followed by other/not reported (20.0%), Asian (4.0%), and mixed race (2.0%).

### Human Grader Agreement

The two expert human graders independently evaluated all images and achieved high agreement across prompts, with κ values ranging from 0.634 to 0.707 across the prompts (Table 1). Ground-truth labels were assigned based on biopsy (for melanoma) or at least 6 months of lesion stability on multimodal imaging (for nevi). The final data set included 29 eyes with choroidal nevus and 19 eyes with choroidal melanoma. The human graders had high overall and per-prompt accuracy (ranging from 81.2% to 93.8%), sensitivity (78.6% to 96.2%), and specificity (ranging from 81% to 95.2%) (Table 2).

**Table 1.** Agreement Analysis between Human Graders

Prompt Number	Percentage Agreement	κ
1	81.2	0.654
2	85.4	0.707
3	83.3	0.675
4	81.2	0.653
5	82.4	0.634
6	83.3	0.680

**Table 2.** Human Grader Performance by Prompt

Prompt	Grader	Accuracy	Sensitivity	Specificity
1	1	0.812 (0.667, 0.896)	0.818 (0.690, 0.917)	0.869 (0.750, 0.954)
	2	0.896 (0.771, 0.958)	0.905 (0.807, 0.971)	0.939 (0.859, 1.000)
2	1	0.851 (0.723, 0.936)	0.857 (0.704, 0.964)	0.842 (0.636, 1.000)
	2	0.938 (0.833, 0.979)	0.931 (0.808, 1.000)	0.947 (0.800, 1.000)
3	1	0.851 (0.723, 0.936)	0.857 (0.704, 0.964)	0.842 (0.636, 1.000)
	2	0.787 (0.646, 0.891)	0.786 (0.609, 0.920)	0.789 (0.579, 0.947)
4	1	0.851 (0.723, 0.936)	0.885 (0.727, 1.000)	0.810 (0.606, 0.952)
	2	0.891 (0.783, 0.957)	0.962 (0.852, 1.000)	0.800 (0.579, 0.947)
5	1	0.870 (0.750, 0.956)	0.794 (0.545, 0.950)	0.910 (0.830, 0.971)
	2	0.915 (0.809, 0.979)	0.932 (0.853, 0.988)	0.944 (0.877, 0.990)
6	1	0.870 (0.743, 0.955)	0.888 (0.773, 0.967)	0.912 (0.832, 0.971)
	2	0.936 (0.848, 0.979)	0.947 (0.865, 1.000)	0.952 (0.888, 1.000)

### Performance of MLLMs

Across the six prompts, performance varied notably between the three multimodal language models (MLLMs): ChatGPT, Gemini, and Perplexity (Table 3).

**Table 3.** MLLM Performance by Prompt

Prompt	Model	Accuracy	Sensitivity	Specificity
1	ChatGPT	0.292 (0.146, 0.396)	0.350 (0.222, 0.466)	0.625 (0.484, 0.645)
	Gemini	0.500 (0.333, 0.625)	0.414 (0.330, 0.470)	0.542 (0.500, 0.583)
	Perplexity	0.354 (0.208, 0.479)	0.348 (0.217, 0.493)	0.597 (0.482, 0.606)
2	ChatGPT	0.417 (0.267, 0.542)	0.172 (0.038, 0.321)	0.789 (0.571, 0.944)
	Gemini	0.542 (0.375, 0.667)	0.586 (0.406, 0.760)	0.474 (0.231, 0.700)
	Perplexity	0.479 (0.333, 0.604)	0.241 (0.097, 0.417)	0.842 (0.636, 1.000)
3	ChatGPT	0.375 (0.229, 0.500)	0.138 (0.034, 0.280)	0.737 (0.500, 0.905)
	Gemini	0.583 (0.417, 0.688)	0.690 (0.500, 0.844)	0.421 (0.190, 0.650)
	Perplexity	0.521 (0.375, 0.646)	0.379 (0.207, 0.566)	0.737 (0.500, 0.917)
4	ChatGPT	0.438 (0.292, 0.562)	0.148 (0.036, 0.302)	0.810 (0.611, 0.950)
	Gemini	0.438 (0.292, 0.562)	0.000 (0.000, 0.000)	1.000 (1.000, 1.000)
	Perplexity	0.438 (0.292, 0.562)	0.000 (0.000, 0.000)	1.000 (1.000, 1.000)
5	ChatGPT	0.312 (0.167, 0.417)	0.290 (0.199, 0.376)	0.637 (0.560, 0.702)
	Gemini	0.479 (0.333, 0.604)	0.393 (0.296, 0.484)	0.725 (0.646, 0.796)
	Perplexity	0.375 (0.229, 0.500)	0.325 (0.230, 0.419)	0.660 (0.583, 0.734)
6	ChatGPT	0.312 (0.167, 0.438)	0.270 (0.168, 0.411)	0.622 (0.547, 0.688)
	Gemini	0.708 (0.562, 0.812)	0.603 (0.367, 0.811)	0.782 (0.697, 0.862)
	Perplexity	0.667 (0.500, 0.771)	0.604 (0.362, 0.822)	0.780 (0.686, 0.867)

### Prompt 1—Multiple-Choice Diagnosis

Prompt 1 evaluated MLLMs’ ability to diagnose the ocular pathology from a potential list of diagnoses without additional clinical information. Performance varied widely across models. Gemini had the highest overall accuracy (0.500; 95% CI, 0.333–0.625), followed by Perplexity (0.354; 95% CI, 0.208–0.479) and ChatGPT (0.292; 95% CI, 0.146–0.396). Sensitivity ranged from 0.348 to 0.414, while specificity was highest for ChatGPT (0.625) and lowest for Gemini (0.542).

A misclassification or error analysis revealed that all models demonstrated notable diagnostic biases. ChatGPT frequently misclassified nevi as melanoma (48.1% of errors) or as choroidal hemangioma or choroidal osteoma (Table 4). Gemini showed a tendency to underdiagnose melanoma: 84.2% of melanoma misclassifications were labeled as nevus. Perplexity also mislabeled many melanoma cases as nevus or choroidal hemangioma or choroidal osteoma, while nevus errors were scattered across several incorrect classes.

**Table 4.** Misclassification Summary for Prompt 1

			Misclassification Class, %						
MLLM	Ground Truth	Misclassification, %	AMD	CH	CN	CO	DR	Melanoma	OP
ChatGPT	CN	93.1	0.0	29.6	—	22.2	0.0	48.1	0.0
	Melanoma	36.8	0.0	57.1	28.6	14.3	0.0	—	0.0
Gemini	CN	17.2	60.0	0.0	—	0.0	0.0	0.0	40.0
	Melanoma	100.0	0.0	0.0	84.2	0.0	0.0	—	15.8
Perplexity	CN	62.1	0.0	27.8	—	38.9	5.6	27.8	0.0
	Melanoma	68.4	0.0	30.8	53.8	15.4	0.0	—	0.0

AMD, age-related macular degeneration; CH, choroidal hemangioma; CN, choroidal nevus; CO, choroidal osteoma; DR, diabetic retinopathy; OP, other pathology.

These patterns indicate that ChatGPT leaned toward overdiagnosis of melanoma, while Gemini and Perplexity leaned toward underdiagnosis, favoring benign diagnosis.

### Prompts 2, 3, and 4

Prompts 2 and 3 evaluated the ability of MLLMs to diagnose the ocular images as either nevus or melanoma without and with clinical information, respectively. In prompt 2 (melanoma vs. nevus), Gemini again demonstrated the highest accuracy (0.542) and sensitivity (0.586), while Perplexity had the highest specificity (0.842).

In prompt 3 (binary classification with clinical information), adding clinical data improved accuracy and sensitivity for Gemini and Perplexity. Gemini achieved the highest accuracy (0.583) and sensitivity (0.690).

Prompt 4 evaluated the ability of MLLMs to diagnose the lesion based on strict clinical guidelines. Performance on prompt 4 (COMS criteria) remained identical across models (accuracy: 0.483), with Gemini and Perplexity showing perfect specificity (1.000) but zero sensitivity.

Misclassification analysis was not performed for prompts 2, 3, and 4, as responses were limited to two classes and are fully characterized by sensitivity and specificity metrics in Table 3.

### Prompt 5—Treatment Recommendation Without Clinical Information

Gemini again had the highest accuracy (0.479), followed by Perplexity (0.375) and ChatGPT (0.312). However, all models showed significant misclassification patterns favoring radiotherapy (Table 5).

**Table 5.** Misclassification Summary for Prompt 5

			Misclassification Class, %		
MLLM	Ground Truth	Misclassification, %	Enucleation	Observation	Radiotherapy
ChatGPT	Enucleation	100.0	—	0.0	100.0
	Observation	86.2	0.0	—	100.0
	Radiotherapy	26.7	0.0	100.0	—
Gemini	Enucleation	100.0	—	0.0	100.0
	Observation	62.1	0.0	—	100.0
	Radiotherapy	20.0	0.0	100.0	—
Perplexity	Enucleation	100.0	—	25.0	75.0
	Observation	75.9	0.0	—	100.0
	Radiotherapy	26.7	0.0	100.0	—

ChatGPT misclassified 100% of enucleation and observation cases as radiotherapy. Gemini misclassified 100% of enucleation and 62.1% of observation cases as radiotherapy. Perplexity misclassified 75% of enucleation and 100% of observation cases as radiotherapy.

### Prompt 6—Treatment Recommendation With Clinical Information

Prompt 6 yielded the best performance overall. Gemini achieved the highest accuracy (0.725), followed by Perplexity (0.647) and ChatGPT (0.314). Gemini's accuracy and specificity were significantly higher than ChatGPT's after applying the Bonferroni correction (*P* < 0.005), and Gemini's accuracy was also significantly better than Perplexity's (*P* < 0.005).

ChatGPT continued to misclassify all enucleation cases and 87.9% of observation cases as radiotherapy (Table 6). In contrast, Gemini's performance improved: it misclassified only 50% of enucleation cases and 12.1% of observation cases as radiotherapy. Perplexity showed improvement but still misclassified 30.3% of observation cases and 50% of enucleation cases as radiotherapy.

**Table 6.** Misclassification Summary for Prompt 6

			Misclassification Class, %		
MLLM	Ground Truth	Misclassification, %	Enucleation	Observation	Radiotherapy
ChatGPT	Enucleation	100.0	—	0.0	100.0
	Observation	89.7	0.0	—	100.0
	Radiotherapy	29.4	20.0	80.0	—
Gemini	Enucleation	50.0	—	0.0	100.0
	Observation	10.3	0.0	—	100.0
	Radiotherapy	58.8	0.0	100.0	—
Perplexity	Enucleation	50.0	—	0.0	100.0
	Observation	27.6	0.0	—	100.0
	Radiotherapy	41.2	0.0	100.0	—

### Significant Differences Between MLLMs and Graders

Significant differences were noted across the MLLMs and human graders for different prompts, even after applying a Bonferroni correction to correct for multiple analyses. For accuracy in prompts 1 and 2, both human graders were significantly superior to Chat GPT as well as Perplexity, while only grader 2 was superior to Gemini. For accuracy across prompt 3, both human graders were significantly superior to ChatGPT, while only grader 2 was superior to Gemini and only grader 1 superior to Perplexity. For accuracy across prompts 4 and 5, both human graders were significantly superior to Chat GPT, Gemini, and Perplexity. Finally, ChatGPT performed significantly worse than Gemini, Perplexity, and both human graders in terms of accuracy for prompt 6 (Table 7).

**Table 7.** Significant Differences for Accuracy and Sensitivity across Various Prompts

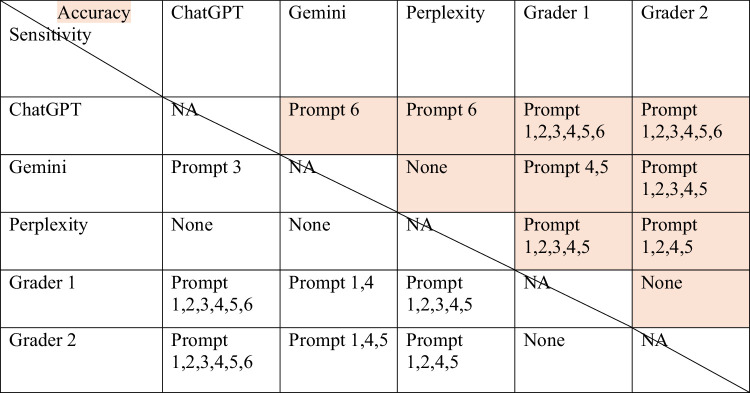

NA, not applicable.

The shaded areas stand for Accuracy, and remaining areas stand for Sensitivity.

Similarly, both human graders had significantly better sensitivity compared to all MLLMs for prompts 1 and 4, significantly better sensitivity than ChatGPT and Perplexity for prompts 2 and 5, and better sensitivity compared only to ChatGPT for prompts 3 and 6.

For prompt 5 sensitivity, Gemini performed similarly to grader 1 but worse than grader 2. Finally, grader 1 performed significantly better than Perplexity, and Gemini was significantly better than ChatGPT for prompt 3 sensitivity (Table 7).

For specificity, there were no significant differences between prompts 3 and 4 across any of the MLLMs or human grader performance. Gemini had worse specificity compared to both graders for prompt 1 and worse specificity than grader 2 for prompts 2 and 5. Both human graders were significantly better than ChatGPT and Perplexity for prompt 5 specificity. ChatGPT was significantly worse than both graders and Gemini for prompt 6 and worse than grader 2 for prompt 1 specificity. Finally, Perplexity performed significantly worse than grader 2 for prompt 1 specificity.

### Intragrader Agreement between Prompts 2 and 4

Since the same eyes were graded in two different contexts by each grader, we evaluated the intragrader discrepancy between the grading done for prompts 2 and 4 for each human grader as well as each MLLM.

• Grader 1:
  ◦ Agreement = 100.0%
  ◦ κ = 1.000
• Grader 2:
  ◦ Agreement = 79.2%
  ◦ κ = 0.585
• ChatGPT:
  ◦ Agreement = 97.9%
  ◦ κ = 0.929
• Gemini:
  ◦ Agreement = 43.8%
  ◦ Perplexity:
  ◦ Agreement = 79.2%

Gemini and Perplexity were unable to produce a κ score, because they both only predicted “Melanoma” for prompt 4.

## Discussion

This study evaluated the diagnostic and treatment performance of three MLLMs—ChatGPT-4, Gemini, and Perplexity—on multimodal ophthalmic imaging data across a structured set of clinical prompts.

Our results echo prior studies demonstrating the limitations and future potential of MLLMs in clinical diagnosis.^19^^,^^20^ ChatGPT consistently underperformed across all prompts, particularly for treatment planning, which may reflect limitations in its integration of multimodal input. A study by Horiuchi et al.^21^ compared the diagnostic performance of text-based ChatGPT-4 versus image-based ChatGPT-4V on skeletal radiology cases. They found that ChatGPT performed better with text-based image descriptions combined with clinical information, whereas providing the image itself with clinical data resulted in poorer performance.^22^ This may indicate that the model currently struggles with interpreting raw visual data, which is consistent with previous studies.^21^^,^^23^ Furthermore, the consistently poor sensitivity for COMS-based decisions (prompt 4) across all models suggests that AI systems may not yet be reliable in applying strict, specialized diagnostic guidelines or standards used by clinicians in their field. To evaluate the flexibility of each grader in adapting to stricter diagnostic criteria, we compared intragrader agreement between prompt 2 (melanoma versus nevus) and prompt 4 (diagnosis based on COMS criteria). This analysis aimed to determine how consistently each grader modified their responses when explicitly instructed to apply image-based guidelines. Gemini and Perplexity generated only “Melanoma” responses for prompt 4, resulting in zero variability and precluding calculation of Cohen's κ score, which requires variability across categories. This suggests that grader 1 and ChatGPT were highly consistent in their responses, regardless of the diagnostic prompt, indicating limited responsiveness to changes in instruction. In contrast, grader 2 showed moderate adaptability, while Gemini demonstrated the greatest flexibility in adjusting its diagnoses to follow COMS-based image criteria. Although Perplexity could not generate a κ score due to uniform responses in prompt 4, its readiness of changing diagnoses was similar to that of grader 2.

Recent work by Zabor et al.^24^ and Singh et al.^25^ developed and externally validated a machine learning model to predict the likelihood of small choroidal melanocytic tumors being melanoma, achieving high discrimination (area under the curve ∼0.86–0.88). Their model incorporates clinical and imaging variables, such as lesion height, subretinal fluid, and orange pigment, and has been translated into a user-friendly online risk calculator for clinical decision-making. Furthermore, their risk stratification approach allows identification of low-risk tumors that can be safely observed without immediate treatment, reducing unnecessary vision loss without increasing metastatic risk. This model was subsequently validated in clinical practice, demonstrating strong predictive performance and clinical utility in guiding management decisions. Their approach, which incorporates key clinical and imaging features, complements our findings by illustrating the growing role of AI-driven tools in improving diagnostic accuracy and personalized care in ocular oncology.

Studies comparing the different MLLMs in relation to medical diagnosis have shown different results in the past, with ChatGPT usually performing better than other models.^26^^–^^30^ In our study, however, Gemini consistently demonstrated superior performance across most prompts, especially when clinical data were included. Perplexity, followed by ChatGPT, demonstrated the poorest performance overall. It is notable that the prior studies focused more on text-based prompts for MLLMs in contrast to our study, which incorporated multimodal imaging. This may suggest that Gemini may be more capable of integrating multimodal inputs for diagnosis and treatment decisions.

Misclassification analyses for treatment prompts revealed a systematic tendency for all models to default to radiotherapy, including for cases that required enucleation or observation. This may reflect the models’ bias toward middle-ground or commonly discussed treatments, which could lead to undertreatment or overtreatment recommendations if used naively in clinical settings. Identifying such systematic errors is critical for evaluating model readiness for real-world deployment. While encouraging, systematic misclassification patterns and variability between models underscore the need for continued evaluation, fine-tuning, and cautious integration of these tools into clinical workflows.

One limitation of our study is the relatively small sample size, which may limit the generalizability of our findings. However, the inclusion of cases with a confirmed diagnosis, coupled with the availability of high-quality multimodal imaging, strengthens the validity and depth of our analysis. The progression of prompts, starting with a broader differential diagnosis, followed by a binary diagnosis, and culminating in a diagnosis based on clinical information, further contributed to the structure and reliability of the study. Another important limitation is the cross-sectional nature of the study; we did not assess the longitudinal evolution of lesions initially diagnosed as nevi that later progressed to melanoma. This would be of interest in future studies to understand how MLLMs integrate longitudinal findings in decision-making. An additional limitation of our study is the use of a 6-month follow-up period to confirm the stability of presumed nevi. While this duration has been used in prior studies, other authors have required 24 months or more, which may provide greater certainty in excluding slow-growing melanomas. A final limitation lies in the diagnostic framework itself: the imaging and clinical features commonly used to differentiate nevi from melanoma primarily indicate the likelihood of growth rather than malignancy. While growth can suggest malignancy, growth rate may be more informative. A study by Harbour et al.^22^ showed that if class 2 tumors were separated from class 1 tumors, with only class 2 tumors being “true melanoma” having metastatic potential, none of the conventional imaging characteristics used to identify melanoma showed statistically significant predictive value. This relates to the imaging criteria being used in prompt 4 (COMS criteria) in our study, where human graders and AI were asked to diagnose the lesion as nevus or melanoma based on the physical lesion characteristics. While the COMS criteria may suggest a higher likelihood of growth, they do not necessarily translate to a definitive diagnosis of melanoma, as growth does not equate to malignancy. Even expert human graders may prefer a “wait for growth” approach unless the lesion presents with unequivocal high-risk features. It is common practice to monitor for growth because many studies have shown how imaging and clinical features can help predict transformation. Regardless, our findings provide meaningful insights into the capabilities and current limitations of MLLMs in ocular oncology, and they emphasize the importance of evaluating their reliability, clinical reasoning, and potential utility in supporting ophthalmic diagnostics.

Numerous studies have evaluated MLLMs using preexisting clinical scenarios from the literature, often with a single-image input.^21^^,^^23^^,^^29^^,^^31^^–^^33^ In contrast, our study is distinctive in its use of disease-specific multimodal imaging acquired from our clinics, and so cases were presented that would not be available for training online. This approach closely mirrors real-world clinical scenarios and diagnostic dilemmas. For instance, differentiating between choroidal nevus and melanoma, particularly with the availability of multimodal imaging, is an important and still difficult task that optometrists and ophthalmologists regularly face in practice. While we evaluated the universally available AI models, research on developing trained AI models specifically for predicting choroidal nevus transformation into melanoma has shown promising results.^34^

In conclusion, our study demonstrates that current MLLMs show variable performance in diagnosing and managing choroidal lesions based on multimodal ophthalmic imaging. Model performance improves with the inclusion of structured clinical information for certain MLLMs, suggesting an alternative integration model for decision-making. As MLLMs continue to evolve, they are likely to continue to improve and may prove useful for ophthalmic providers in decision-making, although their use will need to be carefully implemented to account for safety and to ensure confidentiality concerns are addressed.

## Supplementary Material

Supplement 1
