# Supplementary material for: Assessing the Clinical Utility of Multimodal Large Language Models in the Diagnosis and Management of Pigmented Choroidal Lesions
Source: Transl Vis Sci Technol. 2025 Oct 14;14(10):13. doi: 10.1167/tvst.14.10.13 (PMC12530446; doi:10.1167/tvst.14.10.13)
Supplement: Supplement 1 [file tvst-14-10-13_s001.pdf]

<table>
<tr><td>PROMPT 1: Multiple Choice Diagnosis</td><td>Answer the following question as an ophthalmologist. Assume that you do not know any background information about the patient such as age, sex, or ethnicity. Examine this set of images from the same patient, consisting of an ultra-wide field pseudocolor fundus photograph, fundus autofluorescence, and an ocular coherence tomography scan. Based on these images, which of the following options is the single most likely diagnosis for this patient?
1. Age-related macular degeneration (AMD)
2. Choroidal nevus (CN)
3. Diabetic retinopathy (DR)
4. Choroidal hemangioma (CH)
5. Choroidal osteoma (CO)
6. Melanoma
7. Congenital hypertrophy of the retinal pigment epithelium (RPE)
8. Retinal tear (RT)
9. Other Pathology (OP)</td></tr>
<tr><td>PROMPT 2: Melanoma vs. Nevus</td><td>Answer the following question as an ophthalmologist. Assume that you do not know any background information about the patient such as age, sex, or ethnicity. Examine this set of images from the same patient, consisting of an ultra-wide field pseudocolor fundus photograph, fundus autofluorescence, and an ocular coherence tomography scan. Based on these images, is the pigmented lesion more likely to be a choroidal nevus or a melanoma?
1. Choroidal nevus (CN)
2. Melanoma</td></tr>
<tr><td>PROMPT 3: Melanoma vs. Nevus with Clinical Information</td><td>Answer the following question as an ophthalmologist. Given this set of images from the same patient, consisting of an ultra-wide field pseudocolor fundus photograph, fundus autofluorescence, and an ocular coherence tomography scan, and the patient's symptoms, best-corrected visual acuity (BCVA), age, sex, and ethnicity**, is the pigmented lesion more likely a choroidal nevus or a melanoma?**
1. Choroidal nevus (CN)
2. Melanoma</td></tr>
<tr><td>PROMPT 4: COMS Criteria</td><td>Answer the following question as an ophthalmologist. Evaluate this set of ultra-wide field pseudocolor fundus photograph, fundus autofluorescence, ocular coherence tomography scan, and ultrasound images of the same patient’s eye. Based on the Collaborative Ocular Melanoma Study criteria for choroidal nevus transformation into melanoma[10,11] (below), **is the pigmented lesion likely to be a choroidal nevus or a melanoma?**
<table>
<tr><th>Criterion</th><th>Imaging Modality</th></tr>
<tr><td>Lesion diameter >5mm</td><td>Ultra-wide field pseudocolor fundus photo</td></tr>
<tr><td>Orange pigment</td><td>Fundus autofluorescence</td></tr>
</table>
</td></tr>
</table>

	Subretinal fluid as cap over pigmented lesion	Ocular coherence tomography scan
	Lesion thickness >2mm	B-scan or A-scan ultrasound
	Acoustic hollowness	B-scan ultrasound
PROMPT 5: Treatment	Answer the following question as an ophthalmologist. Evaluate this set of ultra-wide field pseudocolor fundus photograph, fundus autofluorescence, ocular coherence tomography scan, and ultrasound images of the same patient's eye. **Given the diagnosis of choroidal nevus or melanoma, what one treatment would you recommend for this patient?**<br>1. Observation only<br>2.Radiotherapy<br>3. Enucleation	
PROMPT 6: Treatment with Clinical Information	Answer the following question as an ophthalmologist. Evaluate this set of ultra-wide field pseudocolor fundus photograph, fundus autofluorescence, ocular coherence tomography scan, and ultrasound images of the same patient's eye. **Given the diagnosis of choroidal nevus or melanoma, and the patient's symptoms, best-corrected visual acuity (BCVA), age, sex, and ethnicity, what one treatment would you recommend for this patient?**<br>1. Observation only<br>2.Radiotherapy<br>3. Enucleation	

Supplemental table- List of prompts given to human graders and MLLMs
